# Intradural Spinal Cord Stimulation: Performance Modeling of a New Modality

**DOI:** 10.3389/fnins.2019.00253

**Published:** 2019-03-19

**Authors:** David J. Anderson, Daryl R. Kipke, Sean J. Nagel, Scott F. Lempka, Andre G. Machado, Marshall T. Holland, George T. Gillies, Mathew A. Howard, Saul Wilson

**Affiliations:** ^1^NeuroNexus Technologies, Ann Arbor, MI, United States; ^2^Department of Biomedical Engineering, University of Michigan, Ann Arbor, MI, United States; ^3^Center for Neurological Restoration, Cleveland Clinic, Cleveland, OH, United States; ^4^Department of Neurosurgery, University of Iowa Hospitals & Clinics, Iowa City, IA, United States; ^5^Department of Mechanical and Aerospace Engineering, University of Virginia, Charlottesville, VA, United States

**Keywords:** spinal cord stimulation, intradural, modeling, power efficiency, fiber targeting, selectivity

## Abstract

**Introduction:** Intradural spinal cord stimulation (SCS) may offer significant therapeutic benefits for those with intractable axial and extremity pain, visceral pain, spasticity, autonomic dysfunction and related disorders. A novel intradural electrical stimulation device, limited by the boundaries of the thecal sac, CSF and spinal cord was developed to test this hypothesis. In order to optimize device function, we have explored finite element modeling (FEM).

**Methods:** COMSOL^®^Multiphysics Electrical Currents was used to solve for fields and currents over a geometric model of a spinal cord segment. Cathodic and anodic currents are applied to the center and tips of the T-cross component of the electrode array to shape the stimulation field and constrain charge-balanced cathodic pulses to the target area.

**Results:** Currents from the electrode sites can move the effective stimulation zone horizontally across the cord by a linear step method, which can be diversified considerably to gain greater depth of penetration relative to standard epidural SCS. It is also possible to prevent spread of the target area with no off-target action potential.

**Conclusion:** Finite element modeling of a T-shaped intradural spinal cord stimulator predicts significant gains in field depth and current shaping that are beyond the reach of epidural stimulators. Future studies with *in vivo* models will investigate how this approach should first be tested in humans.

## Introduction

All commercially available spinal cord stimulators in clinical use at present are intended for implantation in the epidural space. That is, the electrode lead or array is positioned dorsal to the dura matter that forms the thecal sac containing the spinal cord and the intervening layer of CSF. While some of the original stimulator arrays were inserted intradurally, the epidural space proved simpler to access, and avoided CSF leakage. Taken together, these and other clinical and technical advantages (Gibson-Corley et al., 2014) have driven the field to the present paradigm.

There are currently as many as 50,000 epidural devices implanted annually worldwide (American Association of Neurological Surgeons, 2018). Decades of intense industrial activity and system refinements (Levy, 2013) sparked this change with many patients being the beneficiaries of this progress (Taylor et al., 2014; Kapural et al., 2016). Even so, there are fundamental limitations to the epidural approach that prevent selective neuromodulation of deeper fiber tracts beyond a thin superficial layer of spinal cord. This is in sharp contrast to the direct interface that exists between deep brain stimulator electrodes and neuromodulation targets within the brain. As a result, the capacity of conventional spinal cord stimulators to selectively deliver current to the spinal cord has largely reached a plateau (Zhang et al., 2014). This stimulus delivery limitation may contribute in part to the observation that many patients do not achieve relieve of their symptoms or experience loss of therapeutic effect over time (Hayek et al., 2015; Geurts et al., 2017). Because of these limitations of epidural SCS, our group has explored intradural stimulation as a means of achieving selective, high-efficacy neuromodulation of fiber tracts deep within the spinal cord in order to more effectively treat patients with neuropathic pain (Reddy et al., 2018), visceral pain (Nagel et al., 2018b), and spasticity (Nagel et al., 2017).

In its original conception, the electrode array of our intradural device, termed the I-Patch, was designed to rest directly on the pial surface of the spinal cord (Howard et al., 2011a; Song et al., 2013). The position was gently stabilized by a compliant configuration of lead loops (Oliynyk et al., 2013) that traversed the dura and were anchored to the laminectomy defect (Dalm et al., 2016). Human-scale prototypes were built and their biomechanical performance characteristics were evaluated using both *in vitro* anthropomorphic spinal cord surrogates (Howard et al., 2011b; Oya et al., 2012; Wilson et al., 2012) and *in vivo* large animal (ovine) models (Gibson-Corley et al., 2012; Oya et al., 2013; Safayi et al., 2014). The design parameters were derived from rigorous assessments of the spinal cord geometries across a large number (*n* = 50) of patients (Viljoen et al., 2013a). This was done to insure that the implanted stimulator array would remain fixed to the pial surface of the cord as it moves within the thecal sac during flexion and extension of the back (Viljoen et al., 2014). The mechanical robustness of the device was also investigated by experiment (Viljoen et al., 2013b) and FEM (Grosland et al., 2014). In parallel, SSEPs were recorded in sheep during acute SCS experiments to confirm and quantify the potential advantages of the approach. The findings included post-presentation persistence of stimulation-induced effects (Flouty et al., 2012) and significantly reduced voltage thresholds for evoking SSEPs as compared with epidural stimulation (Flouty et al., 2013). This work culminated in the development of an ovine model of moderate spinal cord injury capable of serving as a test bed for quantifying the response to intradural SCS therapy of animals with mild spasticity (Safayi et al., 2015). Taken collectively, our preliminary data suggested that it would be technically feasible to create a device that could be safely positioned on the surface of the spinal cord and directly modulate targeted spinal cord neural pathways. Explicit advantages of this approach included increased selectivity of deeper neural fibers and a significant reduction in the pulse generator’s power requirements (Dalm et al., 2014).

Ultimately, in order for a new medical device to achieve a substantial impact on public health, many factors must be considered beyond the single issue of potential efficacy. These include cost, ease of use, and the perceived risk vs. benefit ratio of a new device and implantation procedure. For example, when surgeons perceive existing SCS devices as being moderately effective, they will be hesitant to adopt a new device that in theory will be substantially more effective but will require a longer, more technically demanding implantation procedure associated with increased risks. The original I-Patch (IP1) fell into this category because the electrode array was placed directly on the spinal cord surface, and the dural closure technique was technically demanding and did not achieve an immediate watertight seal. The present report describes a second-generation I-Patch (IP2) that is designed to capture the stimulus delivery benefits of an intradural device, without the limitations of increased procedure time and risk associated with the IP1. The IP2 achieves these objectives through design features that enable intradural implantation and the creation of an immediate watertight dural seal using minimally invasive surgical techniques, and with procedure times and risk that are comparable to that of a standard paddle lead stimulator. We anticipate that the implementation of this approach will result in several advantages, including (1) the elimination of risk of lead migration because the electrode array is secured to the dura, (2) no blockage of intrathecal CSF flow because of the thin profile of the intradural component, and (3) much improved penetration depth and target selectivity of the electrical stimuli delivered to the spinal cord, as discussed in detail below.

**FIGURE 1:**
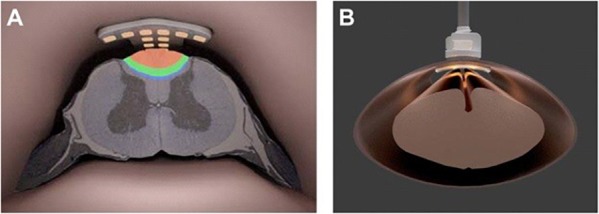
**(A)** Artist’s rendering of the intradural IP2 electrode array projecting current into the dorsal column of the thoracic spinal cord. **(B)** Face-on view of the IP2 array fixed to the spinal dura.

The IP2 device concept is shown in Figure 1. As suggested there, an intradural plate with the stimulator’s electrode array on the distal side has been inserted inside the thecal sac. A hollow threaded stud on the top of the intradural plate extends through the durotomy slot and the overlying extradural plate. A fixation nut is used to secure closure of the durotomy by sandwiching the dura matter between the intradural and extradural plates. Each plate has a gasket consisting of a thin lining of either a dural substitute or some other suitable compressible material on the surfaces contacting the dura, in order to ensure a watertight seal with no significant risk of dural tissue necrosis (Nagel et al., 2019). The leads from the individual electrodes on the intradural array form a bundle that passes through the axial lumen of the threaded stud. The device is secured in place using standard epidural stimulator anchoring techniques, providing stress relief for the lead bundle to ensure that the electrode array remains suspended stably above the spinal cord and does not make contact with it. At its proximal end, the lead bundle is connected to the system’s implantable pulse generator (not shown), used to create and control the stimulation montages.

Our goal here is to present the results of a detailed FEM effort that will optimize the design and location of the intradural electrode array of the hybrid device. This will enable maximal selectivity when targeting of neural fibers within the spinal cord. In particular, we demonstrate that selectivity and depth of stimulation of myelinated nerve fibers in the dorsal columns can be improved by bipolar or tripolar currents emanating from sites on the cross of a T-shaped configuration of electrodes (termed the “T-Array” or “T-Patch”) on the intradural array. Selectivity and depth can be accentuated by lowering the array closer to the dorsal columns (i.e., deeper within the CSF) and perhaps scaling the array down in size to be more specific to the central portion. Additional benefits can be derived by incorporating epidural stimulation sites into the strategy as well. In general, we anticipate that this approach will be able to package reduced power consumption with an enlarged therapeutic window in an easily deployed device.

In what follows, we provide a brief overview of the important role played by modeling in the design, development and clinical use of spinal cord stimulators, with emphasis on intradural approaches. We then present the electro-mechanical details of the hybrid stimulator that has been the focus of our work and describe the COMSOL Multiphysics^®^representation of it, along with the computational approach used to generate the stimulation patterns of interest within the spinal cord and its environs. The results of the work consist of activation mappings of the targeted fiber populations, estimates of power consumption during stimulation sessions, and establishment of the charge density limits for reversible vs. irreversible tissue damage, all as functions of device configuration, location, and stimulation current levels. We then discuss our findings relative to those of others, explore the implications for implementation of novel modes of intradural stimulation, and lay out a program for future studies that will include validation of the model via experimentation and assessment of the issues to be resolved prior to eventual clinical trials.

## Materials and Methods

Ultimately, the effectiveness of any SCS device depends on its capacity to modulate targeted neural elements selectively within the spinal cord, while at the same time sparing non-targeted structures. At the most elementary level this means controlling or steering the electrical fields generated by the currents delivered from the electrode contacts; this usually involves both spatial and temporal control attributes. The spatial distribution of the field strength and gradient determines which neural elements are affected. Current flow through the axons passing into these fields may be susceptible to exogenously triggered depolarization. The temporal pattern of the fields also will influence the axonal action potentials. From this summated response to the field strength and temporal application emerges the ‘selectivity’ and the desired modulation of neural activity.

Our objective is to show how an intradural stimulation device enhances the effectiveness of field control by its closer proximity to the targeted neural elements and by removing the electrical resistance of the dura from the current path. This will dramatically reduce power and contain diffusion of current flow. To carry out this analysis, we have employed FEM, which has been used extensively over the past 30 years to create quantitative bioelectrical descriptions of SCS (Coburn, 1980; Coburn and Sin, 1985; Holsheimer et al., 1991; Holsheimer and Wesselink, 1997; McIntyre and Grill, 2001; Manola et al., 2007; Hernández-Labrado et al., 2011; Holsheimer and Buitenweg, 2015), including high frequency stimulation (Lempka et al., 2015; Arle et al., 2016) and, in a few cases, intradural stimulation (Howell et al., 2014; Huang et al., 2014).

### Model

COMSOL^®^Multiphysics Electrical Currents is used to solve for electrical fields and currents over axial and transverse segments of the spinal cord deep to the dural membrane. Data and graphics are exported for illustration and use by MATLAB-based programs that reconstruct complex fields and simulate the effect of the fields on axons of various sizes within the dorsal column.

### Geometry

**FIGURE 2:**
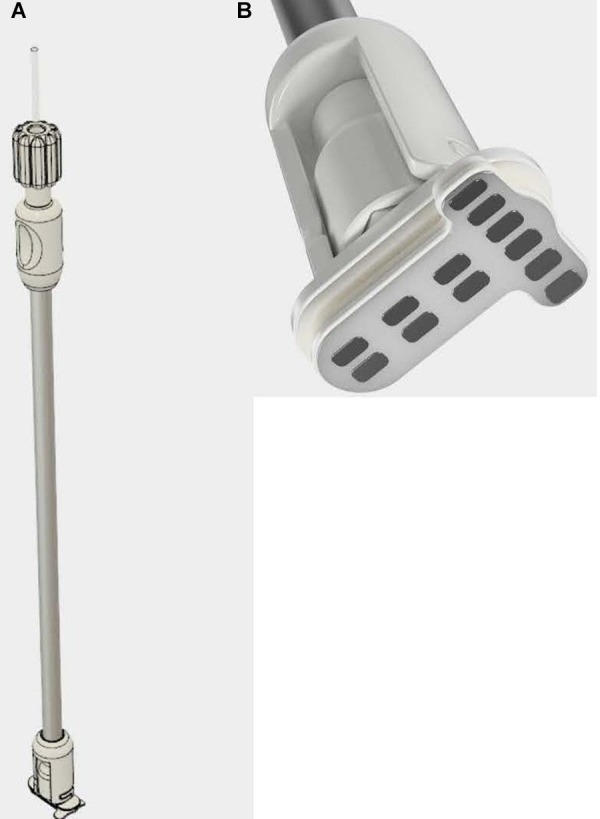
Three-dimensional renderings of prototypes of **(A)** the intradural stimulator implantation tool, and **(B)** the T-shaped intradural electrode array on the distal end of the implantation tool prior to insertion.

Figure 2A,B are renderings of the present version of the implantation tool and intradural stimulator. The device design as shown there reflects the results of careful studies of several different electrode-array arrangements, with the final version optimized for performance in terms of minimizing the overall surface area of the array vs. maximizing the targeting specificity during stimulation. After the dura is opened, the surgeon uses the tool to insert the electrode array within the thecal sac. The inner shaft of the tool gently tightens a closure nut onto the surface of the extradural compression plate. This clamps the dura between the gasket materials on either side of the compression plates and secures the electrode array in place. The outer shaft of the implantation tool is then rotated to release it from the opposing tongue-and-groove joints on the extradural plate, and removed. The lead bundle is connected to one channel of the pulse generator, thus completing the procedure.

**FIGURE 3:**
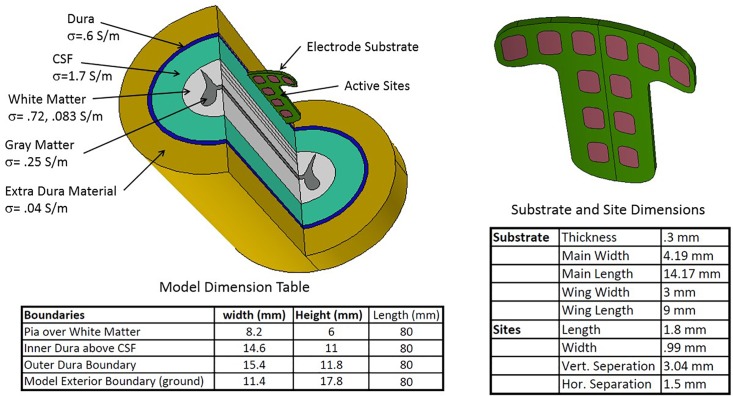
Geometric configuration, conductance parameters, and dimensions of the spinal cord and T-shaped electrode array used in the modeling study.

The scale and contour of the intradural electrode array is matched to that of the adult spinal cord at vertebral levels T8 to T10, which would be the typical locational range for the therapeutic applications discussed later. The features of the human spinal cord captured in geometry for computational purposes are shown in Figure 3. For our simulations, these included (1) a gray matter core, (2) white matter surrounding the gray matter, (3) a CSF layer bounded by the spinal cord and the dura, and (4) the dura itself, which is the outer layer of the model and includes both the dural layer and the extra-dural fat. The boundary condition simulating the dura is a conductive layer which allows current to exit the model and pass to ground. This is not a detailed geometry of the external environment, but it does account for shunting of some internal electric currents, thus reducing the current projected to the excitable tissue and affecting the neural thresholds. The intradural device provides a much higher impedance path for current to enter the extradural space than the traditional paddle positioning.

Also per Figure 3, an electrical conductivity was assigned to each volume. All the conductivities are scalars apart from that of the white matter, which has different longitudinal and radial conductances. Electrical continuity is assumed between each volume. While the volume outside the dura is complex, we are modeling the extradural volume with a single low conductivity material grounded at its outer surface. The cross section of the cord was held constant over the 80 mm of cord simulated. Dimensions, biophysical and electrical quantities are within the range found in the spinal modeling literature (Coburn and Sin, 1985; Holsheimer, 2002; Nagel et al., 2018a), which provides the generally accepted properties of the relevant intradural structures. The substrate holding the 12 sites is positioned just beneath the dura and projects 0.3 mm below the dura in the CSF space. The electrode substrate is not conductive but there is some current leakage beyond the edges of the substrate into the dura and the external space.

### Internal Physics and Boundary Conditions

The continuity condition for zero charge creation (∇σ∇V = 0) holds everywhere in the model interior, all surfaces at the ends of the cord have zero potential (*V* = 0), the outer elliptical surface representing the extra-dural space is conductive and grounded. The sites are current sources such that: $\int_{\partial \Omega }J•nds=I_{0}$. The actual distribution of the current over each site is determined by the surrounding electrical environment. *V* is the dependent variable representing the internal scalar potential voltage, σ is the conductivity scalar or tensor.

The geometry and boundary conditions are used to solve the electrical fields. This is done by discretizing the volume of the model with tetrahedral meshing and then employing the finite element method, both of which are implemented within COMSOL^®^.

### Field Evaluation

Several post-processing methods are available within COMSOL^®^to produce important data products from the field solutions. Among these are visualizations of the fields and currents superimposed on the geometry, calculation of quantiles such as maximums, minimums, averages, integrals etc. over points, lines, surfaces or volumes, and export of any of the products.

### Basis Function Method for Field Reconstruction

When performing many serial computations on fields under different drive conditions, it is convenient to use a basis method to reconstruct each new field dictated by new electrode current delivery. This is accomplished by solving the model for each site excited alone with a unity current (1 mA) while the other sites are set to zero current. A portion of the voltage field covering the mostly white matter and adjoining gray matter is transferred to MATLAB^®^using the COMSOL^®^suite of link functions with the voltage of each site. A complex field generated by several sites with different currents can be approximated accurately by superposition of the basis fields scaled by the current from the sites. In a like manner, the voltage on each site is determined and the power calculated. This method was used when computations such as neural simulation were performed using the MATLAB^®^platform outside COMSOL^®^.

### Neural Models

There are several variations of models for the active nodes of axons, the propagation of neural spikes in myelinated axons and how electrical field potentials can initiate them. The minimum construction is a string of nodes consisting of a capacitive membrane populated by simulators of different voltage-controlled channel species for Sodium and Potassium and held at an equilibrium potential by diffusion potentials. The nodes are then capable of an action potential upon sufficient depolarization of the membrane. When the nodes are connected by conductive and perfectly insulated axonal interiors, it is then possible to propagate action potentials from one node to another through depolarization of adjacent nodes by action potential-driven currents.

**FIGURE 4:**
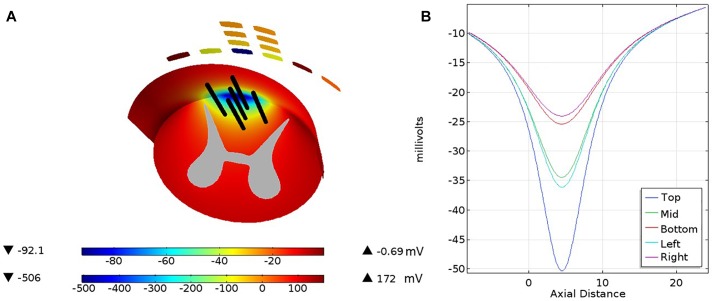
The pia covering of the white matter is viewed from below with a cross section across the white matter and the gray matter cross section. A color coded electrical potential (lower color scale) resulting from a current drive on the cross of the T sites is projected on the pia surface and the white matter cross section (upper color scale). The black lines shown in **(A)** are test lines that are voltage-sampled and displayed in **(B)**.

**FIGURE 5:**
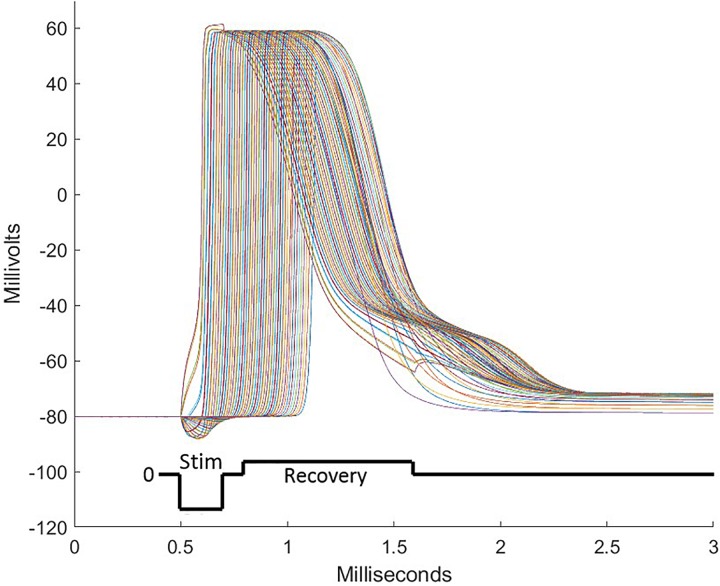
During stimulation pulses, electrode sites over the intended target deliver currents balanced over the several sites of the electrode array. These consist of cathodic depolarizing pulses of short duration on some sites and anodic pulses on other sites, sending some nodes of neurons into action potentials followed by propagation of the action potential in both directions from the initialization. To assure that all sites are individually charge balanced, a recovery pulse is delivered which is opposite in polarity, longer in duration, and lower in amplitude than the stimulation pulse. To achieve the focusing effect to avoid stimulation of off-target tissue, anodic first pulses are delivered peripheral to the central electrode sites. A well-designed pulse complex prevents spread of stimulation from the target area and does not induce off-target action potentials.

The triggering mechanism is the following: electrical stimulation causes initiation of the first action potential by positive second potential differences external to nodes. This will drive the currents causing depolarization of a single node or group of nodes within the influence of a sufficiently strong second difference. Thus, both field strength and field shape are important for initiating action potentials. For example, a constant field potential or a constant potential gradient cannot initiate an action potential in an axon. Figure 4A is a view from under the pia surface. The pia and a cross section plane are colorized with a representation of the potential field. In addition, lines parallel to the axis of the cord are inserted to mark locations of waveform samples shown in Figure 4B. The negative peaking of these spatial waveforms clearly indicate a positive second spatial derivative or difference surrounded by a lesser negative spatial second derivative. Figure 5 shows waveforms of the several axon nodes as several nodes near the electrode substrate are depolarized and ignited into action potentials. Nodes further from the electrode come under the influence of the first to respond and the action potential propagates in both directions from the initiation with nearly identical waveforms but delayed in time.

To visualize neural activation in the model white matter, we create a grid of points in the white matter and adjacent portion of gray matter cross section-spaced 0.1 mm from each other. Each point is populated by three neurons differing in size. The particular axons chosen have node separations of 750, 1250, and 1450 μm corresponding to 7.3, 11.5, and 15 μm diameters, respectively, from Table 1 of McIntyre et al. (2002), which is the MRG model. The calculation proceeds by scanning the grid of points in the white matter by deriving the spatial waveform for that point. Each neuron is tested using the spatial waveform derived for the grid point, resampling to the node spacing and submitting sample points and axon parameters to the MATLAB^®^ODE solver ode45. The solver proceeds with the activation of the test axon with a 200 μs pulse. If any node in the axon passes +5 mV, the solution stops and the axon is recorded as activated. If the smallest axon is activated, the other two are not tested and the grid point is marked with a red x. Similarly, if the medial sized axon is activated, the largest one is not tested and a green x is placed at the grid point. If only the largest axon is activated, a blue x is placed at the grid point. A mark is not placed if no activation is achieved. If activation occurs in the gray matter, a white x is placed on that grid point. These activity cross sections are used in the following figures to indicate excitation within the white matter.

From this minimally complex axon model, several features can be added which increase the fidelity of the simulation. These include better models of the myelinated axon by adding piece-wise cable properties to the conductive segments and more refined populations of channels to the nodes, among others. For instance, the full MRG model separates the membrane/myelin lumped circuit into membrane leakage and capacitance in series with a myelin leakage and capacitance. In addition, a conductive space is added between the membrane and the myelin and this network is often distributed into several networks along the internode. This model adds two differential equations per internode network, which may be repeated as many as ten times. Details in the model matter particularly when investigating complex temporal aspects of stimulation signals but have been suppressed here for simplicity.

## Results

### Tissue Targeting and Selectivity

**FIGURE 6:**
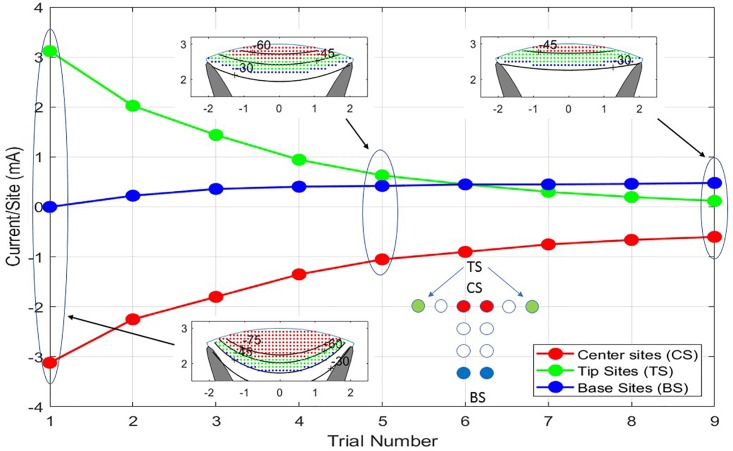
The nine trials shown are a progression from balanced cathodic and anodic currents between the center sites (CS) and the tip sites (TS) of the T cross to balanced currents from the center sites to the base sites (BS) producing different depths of stimulation. As the transition from TS to BS is progressing, all the currents are being scaled down to prevent excitation of neurons outside the white matter. In Trial No. 1, a large current is flowing between CS and TS causing iso-potential lines of the cross section insert to bow downward at the midline of the model. In Trial No. 9, the iso-potential lines are almost horizontal across the model.

The flow of current from any single site on the implant will spread preferentially in the CSF because it has the highest conductivity of any media in the model, e.g., ≈20 times the lateral component of the white matter conductivity tensor. The equipotential lines in the gray and white matter of the spinal cord will tend to be straight lines therefore cutting across not only the white matter but also the gray matter leading to unintended stimulation of cells in the gray matter. The solution is to excite central sites of the implant with a cathodic potential while exciting lateral sites on the tips of the T with an anodic potential. This limits the cathodic potentials from spreading laterally, thus missing the dorsal horns of the gray matter. Figure 6 illustrates this for nine different current levels in the central two sites of the T. As the current from the central sites is diminished, the balancing currents contributed from the tip sites and the base sites are not only reduced to match but also re-proportioned to favor the base sites.

**FIGURE 7:**
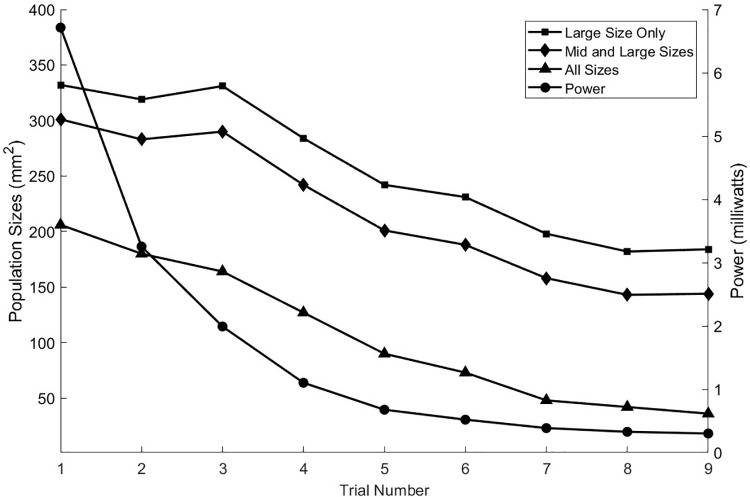
As seen in the previous figure, the depth of stimulation in the white matter can be controlled while preventing unintended stimulation of the gray matter. This is achieved by increasing the anodic currents at the tip sites in greater proportion to the increases in cathodic currents at the central sites. That process can also be viewed in terms of the activation levels of the different sized axon populations as shown here for the nine stimulation trials. The power consumption increases approximately quadratically with the increase in the activities of the three axon classes.

The neural activities shown in the cross sections of Figure 6 are expressed in Figure 7 as areas consisting of large diameter neurons only (squares), middle and large sizes (diamonds), and all sized neurons (triangles). As the anionic current stimulation progresses from all tip sites to all base sites during the nine-step sweep from the left dorsal horn to the center, the areas of all neuron classes decrease somewhat uniformly. The trace with black circles shows that the total power for each trial experiences a dramatic quadratic reduction from maximum tip site involvement to minimal tip site involvement. Note that the reduction of total neural activation does not begin to decrease until the fourth trial, indicating that a considerable power saving (about 75%) can be obtained for the deepest activity profile by involving the base sites.

**FIGURE 8:**
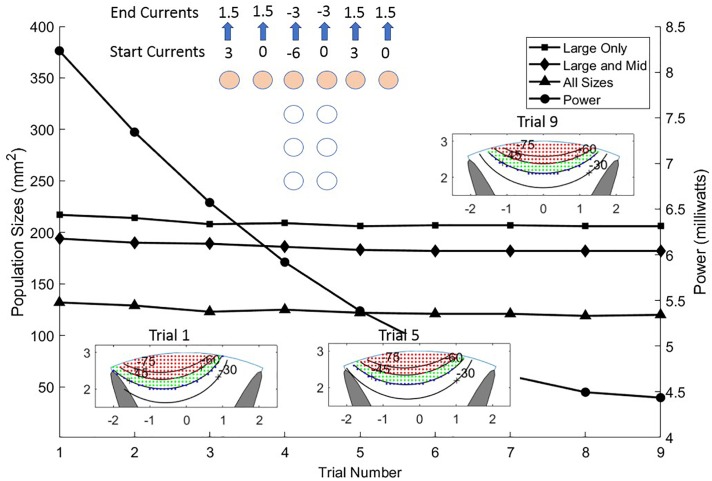
Sliding the stimulus profile across the cross of the T allows selecting different populations of axons laterally across the upper layers of the white matter. This is accomplished by making a linear sweep of current values coming from the six sites of the T cross as shown in the top center of the Figure. Almost identical activation profiles for all three sizes of neurons are seen to progress across the cord from left to center as shown in the three colored graphics and the area curves. The power (circles) is shown for each trial diminishing from left to center to right as current is distributed more evenly over the six sites on the T cross.

As with achieving greater stimulation depth, currents from sites on the T can be manipulated to move the effective stimulation zone horizontally across the cord. Figure 8 illustrates how sweeping of current sources across the T can position the center of stimulation with high spatial resolution. Nine steps are shown but more instances can be placed within the progression thus creating an increased resolution of position. The linear step method utilized to achieve progression of the stimulation center across the cord can be diversified considerably to gain greater depth and perhaps a more skewed pattern by departing from the symmetry of the montage as practiced in the example. The power consumption for the different trials in Figure 8 is not constant but reduces as more sites take part in the stimulation. The first trial uses only three sites, while the ninth uses six sites.

When shaping the stimulation field, we can constrain the cathodic pulse to a target area but the electrochemistry of the electrode sites requires that there be a recovery phase of the stimulus waveform that charge-balances the net stimulation to zero. This means that the tissue volume not included in the target will receive a cathodic current during the recovery phase that may excite neurons in an unintended volume. The method that is usually used makes the recovery current much smaller than the stimulation phase over a longer time. The requirement for this factor in the stimulus design may limit the depth and selectivity strategy in some cases.

### Power Consumption

Electrically bypassing the dura achieves a large power saving due largely to the impedance of the dura with respect to the subdural materials such as the CSF. In addition to the extra voltage drop over the dura, some of the current from the sites leaks out of the dural layer and never reaches the CSF. Depending on the exact geometry and the materials at the interface, additional work now underway indicates that this loss can be in excess of 10% of the current delivered by the sites if the seal to the upper surface of the dura is perfect, and more if there is scar tissue between the dura and the device. This leakage must be made up in extra current through the sites to achieve results comparable to the subdural device. This feature can result in longer battery life. In addition, the subdural device projects slightly (0.3 mm) into the CSF space bringing the electrode sites closer to the excitable elements of the white mater and thus less current spread within the CSF. This better proximity improves the ability to steer or focus the electrical potential within the white matter, but is not evident in this simulation due to the low profile of the device below the dura.

Independent of the reduced impedance barrier and improved proximity, curving of the iso-potentials is needed to obtain selectivity and improved depth. Figure 6 shows improved depth by passing more current through the central sites and through the tip sites on the ends of the T cross. However, the improvement of depth obtained from no involvement of the tips sites on the ends of the T cross to maximal involvement of them requires an 18-fold increase in power. Figure 7 shows that much of this power increase can be mitigated by passing some of the anionic current through the base sites of the electrode array. Any large current places an electrochemical stress on the electrode materials and also on the tissues just under the pia mater. To determine the limits, a safety analysis is required as discussed below.

### Safety Limits

There are two important safety considerations required for long-term sustainability of neural stimulation devices. The primary consideration is the tissue’s tolerance to current density and total charge per pulse-phase, but prevention of electrode failure is also paramount (McCreery et al., 1990; Cogan et al., 2016). Site corrosion can lead to failure of a device and poisoning of the target tissue. The electrode sites used in the IP2 are small by the standards of dorsal column stimulation devices. This is justified by the proximity of the sites to the target tissue and the absence of the impedance and distance barrier presented by the dura. The area of the sites is 1.72 mm^2^ and the accepted safe charge delivery per phase for platinum (Kelliher and Rose, 1987; Grill, 2005) is 50 μC/cm^2^ yielding 0.86 μC as the charge limit per phase for the T-array site. (We note that this value of 50 μC/cm^2^ refers to the threshold for the onset of electrochemical effects for platinum electrodes, and not to the threshold for the onset of neurological tissue damage. The latter has a different value and it is discussed separately below). Thus, for a 200 μs pulse, the largest current allowed is 4.3 mA. We further assume that the capacitance of the electrode’s surface is 0.45 F/m^2^. In principle, materials with higher effective capacitance might also be considered for this application, but platinum is typically chosen because its properties allow for high charge densities with relatively low risk of Faradaic reactions.

**FIGURE 9:**
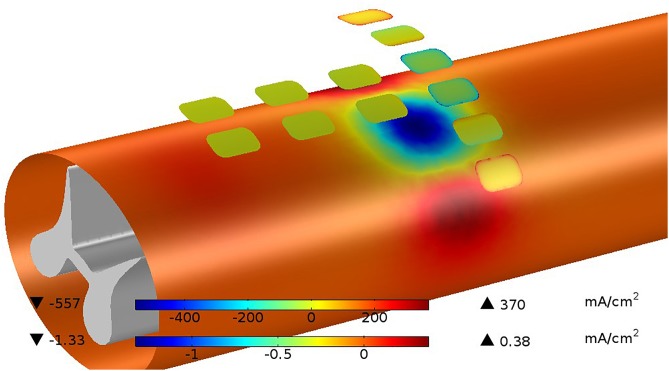
Current distributions are shown for sites and the pia surface of the white matter at the instant the cathodic stimulation phase is started. The current on the site edges (upper color bar) are very large with respect to the average current but this quickly disappears as the charging of the site progresses. The current passing in and out of the white matter (lower color bar) is much less than the current at the sites because of the shunting effect of the CSF. While the total current at the cathodic sites is 4 mA, the current passing into the white matter is only 0.13 mA or 3.25%.

As an example, a pulse sequence consisting of a 200 μs cathodic phase of 2 mA followed by a 400 μs anodic phase at 1 mA uses about half the capacity of the site. The average interface voltage and the drive voltage will be equal as the sequence starts but diverge as the site becomes charged. As the sequence begins, the drive voltage jumps to overcome the spreading resistance then ramps downward due to (1) the increased average charge across the site and (2) to lower efficiency of charge transfer resulting from greater charge at the periphery. This rapid accumulation of charge at the site edge results from increased current flow there. If the sites and pulse protocols are not designed to control edge currents, site corrosion can occur. During the first few microseconds of a pulse, the imbalance between center and edge current can be somewhat extreme, but as time progresses, the current density across the site becomes more even. At the end of the cathodic phase, the potential difference due to extra charge at the edge is about 100 mV. The magnitude of the edge current at the beginning of the pulse can be reduced by several ways. (1) Keeping the curvature of the site perimeter to a minimum is important. Circular sites are the most efficient when used alone. (2) Shaping the pulse’s leading edge to have a longer rise-time allows charge to build up more slowly, thus reducing the maximum current density at the edge (Wang et al., 2014). (3) Preventing adjacent sites from having extreme polarity differences will prevent large currents from flowing directly from site to site. Evenness of this current is promoted by parallel edges of adjacent sites. This is another cost of target selectivity. (4) Adding a resistive layer over the site forces the current flow to be more evenly distributed, but at the cost of additional power because of the resistive voltage drop and a less efficient current flow into the media. Figure 9 shows an example of the current distributions for the sites and the pial surface of the white matter at the instant the cathodic stimulation phase is started. The pial surface current density distribution changes minimally over the charge injection pulse because of the gap between the site and target, but the current distribution on the sites changes considerably as described above.

None of the examples used in this paper exceed the threshold for the onset of electrochemical effects in platinum of 50 μC/cm^2^. Similarly, there are limits of current density that can be tolerated by neural tissue. The maximum current density seen in our maximum depth-of-excitation figure is 1.33 mA/cm^2^, which for the 200 μs pulse is 0.266 μC/cm^2^, well below the commonly accepted boundary of 30 μC/cm^2^ (McCreery et al., 1990; Cogan et al., 2016).

## Discussion

### Comparison With Results of Others

Huang et al. (2014) carried out an early effort to model stimulation profiles of the original version of the I-Patch, in which the electrode array rested directly on the dorsal pial surface of the spinal cord and compared them against epidural stimulation. The COMSOL^®^implementation employed a volume conductor model with domains for the CSF, white matter, gray matter and a pair of electrodes, and the nerve fiber model of McNeal (McNeal, 1976). They found that the current threshold for axonal recruitment in the dorsal columns was over 10 times smaller for direct intradural vs. epidural stimulation, with equally improved depth of stimulation. Moreover, these findings were consistent with the outcome of an *in vivo* study of acute intradural stimulation in an ovine model (Flouty et al., 2013). While pointing the way toward potentially greater therapeutic efficacy, the invasive implantation procedure for a clinically useful device placed directly on the surface of the spinal cord would have carried greater surgical risk than a device placed in the extradural space. The present configuration of the IP2, per the device depicted in Figure 2, addresses this limitation by placing an intradural electrode array flush with the inner surface of the dura, and not in direct contact with the spinal cord.

For purposes of comparison with our findings, those of Howell et al. (2014) are perhaps the most relevant. Their detailed report covered the COMSOL^®^-based modeling and acute clinical testing of intradural SCS, as carried out both epidurally and intradurally with an AD-TECH^®^Spencer Probe Depth Electrode. They found that there was a > 90% reduction in the power needed to activate dorsal column fibers with intradural stimulation relative to epidural stimulation, and that there was likewise a significant improvement in stimulation selectivity. The specific configurations investigated involved having the electrodes (a) 1 mm above the dura matter, (b) 1 mm above the spinal cord, and (c) 1 mm below the dura matter, with the alignments directly above the midline and also at 10 and 20° angular offsets from the midline. The configurations where the electrodes were just below the dura would bear the closest resemblance to the intradural T-array IP2 described here.

Although the vast majority of all the other efforts aimed at modeling SCS have focused on epidural methods, the goals have typically been similar to ours, i.e., to investigate the selectivity of stimulation and optimize the potential for therapeutic benefit. The University of Twente Spinal Cord Stimulation Software in particular has been used extensively to study how different configurations of electrodes and stimulation montages can enable steering of the current distributions (Sankarasubramanian et al., 2011, 2013, 2014). One of the goals is to achieve improved recruitment of dorsal column fibers, which in our case is accomplished by sweeping the current sources across the sites on the T-array and, in general, by the closer proximity of the array to the dorsal surface of the spinal cord.

### Limits of the Study and of the Device Design

The design of our intradural stimulation system incorporates the possibility of using one or more auxiliary epidural electrodes to enable comparisons between montages that are purely epidural, purely intradural or combined in nature. However, since the structure of the implant prohibits any such auxiliary site from being simultaneously positioned directly above the same tissues underlying the intradural array, it has played no role in our modeling effort.

Moreover, while there are several other possible geometrical arrangements for the electrodes of the intradural array, we report results for only the T-shaped version, as it will likely be the easiest to place intradurally of the various alternatives we have considered so far. The version of the T-array modeled here employs 12 electrode sites, whereas most standard paddle leads at present incorporate 16 sites. The need to minimize the array’s surface area is partly responsible for this difference. However, as shown above, the closer proximity to the target fibers, the circumvention of the dura mater, and the unique geometric arrangement of the sites all combine to optimize the selectivity of stimulation targeting. Even with those advantages, a 12 site design may have limited compatibility with some implantable pulse generators requiring 16-line connectors.

### Implications for Improved Therapies

#### Visceral Pain

The depth and control of the stimulation patterns achievable with intradural stimulation suggest that it may be possible to activate a narrow column (1–2 mm wide) of midline pathways perhaps up to 5 mm below the dorsal surface, thus achieving the reversible modulation equivalent of punctate midline myelotomy. The inception of this procedure was first described by Armour (1927) and termed the “commissural midline myelotomy” for the treatment of visceral cancer pain. The surgical goal of the commissural midline myelotomy was to transect the crossing fibers of the anterior lateral sensory system at the level of the patient’s pain. This technique, despite years of refinement, continued to yield an elevated rate of unwanted side effects (Viswanathan et al., 2010). This is believed to be due to the need to access the ventral portion of the spinal cord via a dorsal approach, increasing the likelihood of undesired injury to surrounding spinal tissues. However, basic scientific studies have revealed the presence and importance of the newly described post-synaptic dorsal column visceral pain pathway, lesioning of which likely produces the efficacious benefits of the commissural midline myelotomy. First, the rodent model utilized by Hirshberg et al. (1996) demonstrated the anatomical presence of the post-synaptic dorsal column pathway. In this visceral pain system, the peripheral sensory neurons synapse with dorsal horn neurons within laminae II and IV. The dorsal horn neurons then project axons that ascend ipsilaterally within the dorsal columns and synapse with neurons within the nucleus gracilis or cuneatus. These third-order neurons then decussate in the brainstem and synapse in the thalamus to be relayed to higher order cortical structures. Further physiological studies confirmed that these neurons are activated by visceral pain stimuli (Ness and Gebhart, 1988; Al-Chaer et al., 1996), and a small targeted transverse lesion in the midline dorsal column of the spinal cord resulted in decreased activity in the visceral pain pathway neurons within the ventral-posterolateral nucleus of the thalamus (Al-Chaer et al., 1996).

The localization and physiological characterization of this pathway has led to a modification of the original surgical technique of treating visceral pain described as the “punctate midline myelotomy.” The first reported case of the punctate midline myelotomy involved creating a lesion at the T8 level that completely eliminated a patient’s intractable, residual visceral pelvic pain following treatment of uterine cervical cancer (Nauta et al., 1997). In a later series of five patients performed by the same surgeon a lesion 5 mm in depth and 1 mm on each side of the midline was highly effective in relieving visceral pain symptoms (Nauta et al., 2000). Additionally, a percutaneous midline cervical spinal cord myelotomy technique (Kanpolat et al., 2002) has been used to effectively treat medically refractory visceral pain in patients with advanced malignancies. Currently the myelotomy procedure is used to treat only a small subset of patients with debilitating medically refractory visceral pain; typically patients with end stage malignancies. This restricted use is based on a combination of factors which have limited clinical adoption, the most significant being that the myelotomy is an ablative procedure. Based on the limited clinical series reported to-date, the risk of creating new neurological deficits that can be detected by standard clinical testing appears to be very low. However, lesions of this pathway may cause irreversible disruption of more difficult to quantify functions, such as sexual functions. In patients with end stage terminal illnesses, these risks are outweighed by the benefits of an ablative procedure that is highly effective in reducing or eliminating debilitating visceral pain.

For all patients with medically refractory visceral pain, but in particular for those with a prolonged life expectancy, it would be highly desirable to have the capacity to safely and reversibly modulate the neural pathway that is ablated during the punctate myelotomy procedure. The potential clinical indications for such a device would be expanded dramatically if its use could be extended to patients with non-cancer visceral pain. Chronic abdominal pain from inflammatory conditions is unfortunately common, and treatment modalities are limited. As pain management alternatives to the extensive use of opioids are sought, new technologies such as the present can become increasingly relevant. In addition, if improved modulation of midline pathways proves effective in relieving axial spine pain, as suggested by the results of recent experimental animal studies (Nauta et al., 2018), the relevance of this novel approach to public health will be even greater. The results of the present modeling study demonstrate that the IP2 will be capable of selectively projecting neuromodulatory current to a depth within the dorsal column that fully recapitulates the punctate midline myelotomy procedure (i.e., 5 mm). In contrast, earlier modeling work shows that standard extradural spinal cord stimulators are only capable of selectively modulating the most superficial ∼0.3 mm (<10%) of this pathway (Holsheimer, 2002; Holsheimer and Buitenweg, 2015). Carefully designed IP2 clinical studies will be required to determine how this greater than ten-fold improvement in neuromodulation capacity for the visceral pain pathway correlates with clinical efficacy.

#### Neuropathic Pain

The SCS is commonly used to treat select patients with medically refractory neuropathic pain. A wide range of device design and electrical stimulation parameter concepts have been reported. All of these strategies are designed to inhibit the transmission of neuropathic pain signals by applying an electrical stimulus that disrupts pathologic sensory neuron firing patterns associated with the perception of neuropathic pain. These include standard frequency stimulation protocols that evoke paresthesias, as well as more temporally complex higher frequency approaches designed to achieve paresthesia-free pain relief. Despite its frequent use, there are still limitations related to the implementation of SCS for the treatment of neuropathic pain syndromes; in particular, difficulties in driving stimulation to the dorsal columns without activating nearby dorsal rootlets. The IP2 is designed to accommodate delivery of the full range of electrical stimulation paradigms that are currently in use with extradural SCS devices. The results of this study show that the IP2 device will be capable of delivering these same stimuli to the targeted regions of the spinal cord with markedly improved power efficiency, site selectively, and volume of tissue activation, hence producing more effective stimulation with a lower probability of causing adverse or undesired effects. In addition, recently developed closed-loop sensing and stimulus adjustment technologies might be enhanced by using higher fidelity neural signals recorded from intradural IP2 electrode contacts (Russo et al., 2018).

#### Spasticity Following Spinal Cord Injury

In the past, at small number of centers, extradural spinal cord stimulators were placed to treat patients with medically refractory spasticity. Spasticity resulting from central nervous system injury causes pathologic changes in neural processing within the spinal cord. The rationale for SCS treatment in this clinical setting is that electrical stimulation of certain targeted spinal cord structures may reverse or mitigate these post-injury changes and reduce spasticity. Results from published series were mixed and spasticity is not an approved indication for SCS placement currently (Nagel et al., 2017). Extrapolating from information derived from contemporary neuroscience research regarding the pathophysiology of spasticity, and the promising clinical results observed in some patients implanted with extradural SCS devices, it is possible that the enhanced stimulus delivery capacity of an intradural stimulator may enable a device such as the IP2 to be more consistently effective in relieving the symptoms of spasticity.

### Directions of Future Work

We are presently carrying out a series of pre-clinical tests in a large animal (porcine) model using the prototype device shown in Figure 2. The goal of these studies is to provide evidence of the technical feasibility and safety of the IP2 device and surgical implantation procedure, in support of a future FDA approved first-in-human pilot clinical study. During that pilot clinical study the efficacy and safety of intradural stimulus delivery will be systematically examined.

## Conclusion

The results of the current study quantify the advantages of intradural electrical stimulation using validated stimulation modeling methods. An intradural device such as the IP2 will have the capacity to modulate key therapeutic targets within the spinal cord that cannot be selectively modulated using current devices. A future clinical trial will be required in order to determine how this enhanced stimulus delivery capacity impacts clinical efficacy.

## Author Contributions

DA, GG, SN, MTH, and MAH prepared the manuscript draft with important edits and intellectual input from DK, AM, SL, and SW. All authors approved the final manuscript.

## Conflict of Interest Statement

MAH, SW, MTH, DA, DK, and GG are co-inventors on patents covering the I-Patch intradural spinal cord stimulator described here. MAH, SW, MTH, and GG may receive patent royalties from the commercial license of the I-Patch intradural stimulator’s intellectual properties negotiated by their respective institutions. GG and MAH hold equity in the licensee and serve on its Board of Directors, respectively. SL is a shareholder and scientific advisory board member of Presidio Medical, Inc. AM has distribution rights with Cardionomics and Enspire DBS, which are not related to this technology. He is a consultant with Abbott. The remaining author declares that the research was conducted in the absence of any commercial or financial relationships that could be construed as a potential conflict of interest.

## Abbreviations

CSF: cerebrospinal fluid
FDA: Food and Drug Administration
FEM: finite element modeling
IP: I-patch
MRG: McIntyre–Richardson–Grill model
SCS: spinal cord stimulation
SSEP: somatosensory evoked potential
